# Revisiting perceptual sensitivity to non-native speech in a diverse sample of bilinguals

**DOI:** 10.1016/j.infbeh.2024.101959

**Published:** 2024-05-22

**Authors:** Victoria L. Mousley, Mairéad MacSweeney, Evelyne Mercure

**Affiliations:** a Centre for Brain and Cognitive Development, School of Psychological Sciences, Birkbeck, University of London, London WC1E 7HX, United Kingdom; b Deafness, Cognition and Language Research Centre, University College London, London WC1H 0PD, United Kingdom; c Institute of Cognitive Neuroscience, University College London, London WC1N 3AZ, United Kingdom; d Department of Psychology, Goldsmiths, University of London, London SE14 6NW, United Kingdom

**Keywords:** Bilingualism, Speech perception, Language acquisition, Perceptual narrowing, Visual habituation, Eye-tracking

## Abstract

Werker and Tees (1984) prompted decades of research attempting to detail the paths infants take towards specialisation for the sounds of their native language(s). Most of this research has examined the trajectories of monolingual children. However, it has also been proposed that bilinguals, who are exposed to greater phonetic variability than monolinguals and must learn the rules of two languages, may remain perceptually open to non-native language sounds later into life than monolinguals. Using a visual habituation paradigm, the current study tests this question by comparing 15- to 18-month-old monolingual and bilingual children’s developmental trajectories for non-native phonetic consonant contrast discrimination. A novel approach to the integration of stimulus presentation software with eye-tracking software was validated for objective measurement of infant looking time. The results did not support the hypothesis of a protracted period of sensitivity to non-native phonetic contrasts in bilingual compared to monolingual infants. Implications for diversification of perceptual narrowing research and implementation of increasingly sensitive measures are discussed.

## 1 Introduction

Perceptual narrowing is thought to be a robust and reliable process of human language acquisition (Werker & Tees, 1984). Newborn infants are proposed “universal listeners”, able to discriminate phonetic contrasts in any language (Best & McRoberts, 2003; Eimas et al., 1971; Friendly et al., 2014; Kuhl et al., 2006; Narayan et al., 2010; Streeter, 1976; Trehub, 1976; Werker & Tees, 1984; Werker, 2018). Throughout the first year of life, repeated exposure to native language sounds is thought to drive experience-dependent organisation of infants’ perceptual abilities. More specifically, infants develop and then maintain their sensitivity to native language phonetic contrasts (Kuhl et al., 2006; Werker & Tees, 1984) whilst at the same time, their sensitivity to most (but not all, see Best & McRoberts, 2003) non-native contrasts declines. This developmental evolution is informed by patterns and variability in an infant’s early language environment (Best et al., 2016; Kuhl et al., 2006; Werker & Curtin, 2005). However, the field has focused primarily on trajectories of perceptual narrowing in monolingual children who are learning one language and thus one set of phonetic rules. The intricacies of perceptual narrowing in children learning two languages from birth, who make up a significant portion of the world’s population, has been paid much less attention by comparison (for review, see Byers-Heinlein & Fennell, 2014; Costa & Sebastián-Gallés, 2014; Singh et al., 2022; Werker & Byers-Heinlein, 2008).

Some have theorised that the demands of learning two languages may drive a “perceptual wedge” in young bilinguals, such that they might show a protracted period of perceptual openness to non-native speech compared to monolinguals (Petitto et al., 2012). However, clear behavioural evidence defining the nuances of this potential advantage – its developmental timeline, context-specific constraints, and/or the extent to which it may be sensitive to variability in input – has not yet been developed. The present study compares monolingual and bilingual 15 to 18 month olds and the trajectory of their (declining) ability to discriminate non-native phonetic contrasts. Further, visual habituation paradigms used to test perceptual narrowing hypotheses typically rely on the experimenter to make button-presses to represent the infant’s looking behaviour. This approach can be prone to error and/or bias. In the current study, an eye-tracking method was developed to measure infant looking time objectively and precisely.

The early language environments of monolinguals and bilinguals differ in many important ways. Bilinguals face more phonetic variability overall than monolinguals (Singh & Tan, 2021), and the speech input they receive from adults is more variable than that of monolinguals. For example, bilingual children’s parents may or may not be bilingual themselves and may show a wide range of possible language use patterns. When speaking to their child, bilingual parents might always use one language, whilst others may alternate between their languages depending on the context (e.g., English when with English-speaking friends, French at home). Some bilingual parents speak one of their languages with an accent that could differ phonetically, phonologically, and/or rhythmically from native speakers of the same language. Parents may or may not borrow words across languages or mix their two languages within sentences or sentence to sentence (Byers-Heinlein, 2013, 2015). Some bilingual infants have experiences with many different speakers of each of their languages, whilst others only have one adult that uses one of their languages with them (Gollan et al., 2015). There are many ways to be bilingual, and there is a high degree of variability within the experience of learning two spoken languages.

Regardless of how many languages they learn, all hearing children must develop skills in speech perception to detect the acoustic properties of speech, build native phonological repertoires, differentiate linguistic rhythms, and parse speech streams to identify and learn words. Bilinguals must do all of this in two languages, acquiring two phonological repertoires with two sets of phonetic categories (Bosch & Sebastián-Gallés, 1997), lexicons, and statistical regularities (Byers-Heinlein et al., 2010). Unlike monolinguals, bilinguals must also differentiate between their two native languages to learn them as two distinct systems (Byers-Heinlein et al., 2010; Werker, 2012).

It is also true that a bilingual child’s experience with speech is split to some degree between their two native languages. They have less experience with each of their native languages than a monolingual does with their one. This means that, compared to monolinguals, bilinguals have more to learn, must do so in more variable environments, and have less opportunity to practice (Byers-Heinlein & Fennell, 2014; Hoff, 2013; Werker et al., 2012). Despite this, bilinguals and monolinguals appear to reach early language milestones on similar timelines, particularly when measured as “total” or conceptual linguistic knowledge accounting for both bilinguals’ native languages (vocabulary: Bosch & Ramon-Casas, 2014; native language word segmentation: Orena & Polka, 2019; pitch and tone perception within bilinguals: Singh & Foong, 2012; for review, see Höhle et al., 2020).

All infants must be able to perceive the sounds of their native language(s) that comprise its lexicon. An English-learning infant, for example, must be able to perceive the difference between “/r/” and “/l/” to then use these sounds to segment the English words that contain them. Sensitivity to native phonetic contrasts is one domain under the umbrella of perceptual narrowing (for review, see Flom, 2014; Maurer & Werker, 2013). According to perceptual narrowing, infants’ discrimination abilities for native contrasts are present from birth and are evident throughout the first year of life (Kuhl et al., 1992; Werker & Tees, 1984). Strengths of effects likely vary, particularly in relation to the nature of a bilinguals’ two languages, phoneme pairs tested, and the sensitivity of paradigms used, but this native language trajectory appears to describe both monolinguals and bilinguals. Two studies have found that monolingual and bilingual four month olds showed evidence of discrimination of a native vowel contrast (Bosch & Sebastián-Gallés, 2003; Sebastián-Gallés & Bosch, 2009). Interestingly, in both studies, eight-month-old monolinguals but not bilinguals showed sensitivity to the native contrast (“/e/” – “/ε/”; Bosch & Sebastián-Gallés, 2003; “/o/” – “/u/” and “/e/” – “/u/”; Sebastián-Gallés & Bosch, 2009). When tested on a more distant (“/e/” – “/u/”) native contrast than in the original experiments, bilingual eight month olds did show evidence of discrimination (Sebastián-Gallés & Bosch, 2009). Another study using an anticipatory saccade paradigm suggested that eight-month-old bilinguals were sensitive to a native vowel contrast (Albareda-Castellot et al., 2011), and evidence from French-English bilinguals has also reported native vowel contrast discrimination in bilingual eight month olds (Burns et al., 2007; Sundara et al., 2008). Overall, monolinguals and bilinguals can perceive native phonetic contrasts over the first year of life, at least from the fourth month to the twelfth, though they may also show slight differences in sensitivity around eight months.

The other relevant trajectory of perceptual narrowing is that of infants’ declining perceptual sensitivity to non-native phonetic contrasts, driven by increased native language specialisation over the first year of life. Research with monolingual infants suggests a decline in perceptual sensitivity to non-native vowel contrasts between six and eight months of age (Kuhl et al., 1992; Polka & Werker, 1994) and to non-native consonant sounds between eight and 12 months (Best, 1994; Werker & Tees, 1984). According to these patterns, an infant younger than eight months who is learning only Japanese, with no exposure to English, would be able to distinguish between “/r/” and “/l/”, which are phonetically contrastive in English but not in Japanese. However, by approximately eight months, they would be unlikely to show evidence of sensitivity to the “/r/” – “/l/” contrast. Much less is known about whether bilinguals follow a similar trajectory. This is the focus of the current study.

On the one hand, it could be the case that monolinguals and bilinguals develop along similar non-native trajectory to each other. One theoretical framework for bilingualism posits that bilinguals might acquire two language input systems that they assimilate into one cognitive model for language (for review, see De Houwer, 1995). If bilinguals construct one overarching language system in which they store and retrieve information in either of their two native languages, they may behave similarly to monolinguals on certain tasks. In some behavioural domains, evidence suggests similarities in monolinguals and bilinguals’ lexical development (Pearson et al., 1993, 1995), comprehension of speech (Shook & Marian, 2012), and overall achievement of language milestones (for review, see Byers-Heinlein & Lew-Williams, 2018). A non-mutually exclusive view is that, while monolinguals and bilinguals show similar success in the end goals of language development, there may be subtle differences in the paths they take towards native language specialisation.

Research with tonal languages has generated interesting hypotheses about the different potential paths of monolinguals and bilinguals. Tonal contrasts are minimal pairs based on pitch contours that convey information about the different meanings of lexical items within syllables of the same segmental (e.g., consonant and vowel) structure. Research into the perception of non-native tonal contrasts in monolingual and bilingual infants has shown that both groups appear to show U-shaped trajectories but along different developmental timelines. Young infants seem to show an initial sensitivity to non-native sounds that reduces towards the end of the first year (see also Best, 1994; Kuhl et al., 1992; Polka & Werker, 1994; Werker & Tees, 1984); however, sensitivity could later re-emerge, tracing a “U-shaped” trajectory. In a study with Dutch-learning monolinguals, Liu and Kager (2014) reported evidence of non-native contrast discrimination at five to six months and 17 to 18 months but not at nine months. In a study of bilinguals learning Dutch and another non-tonal language, Liu and Kager (2016) again reported the re-emergence of the ability to discriminate non-native contrastive sounds, but this was observed among bilinguals at 11 to 12 months of age rather than 17 to 18 months as in the monolinguals. In contrast, a similarly designed study with a diverse bilingual sample did not find evidence of group differences between monolingual and bilingual infants’ non-native tonal contrast perception across the first two years of life (Kalashnikova et al., 2023). In sum, there is mixed evidence suggesting that monolingual and bilingual infants differ in their perception of non-native tonal contrasts over the first two years of life. In research investigating consonants as units of speech sound contrasts, it remains unknown what trajectories monolinguals and bilinguals take towards non-native discrimination.

In studies of infants learning non-tonal languages, neurophysiological studies of consonant discrimination have shown different neural responses to non-native consonant contrasts in bilinguals than in monolinguals towards the end of the first year of life (Ferjan-Ramirez et al., 2017). For example, Petitto et al. (2012) found that 10- to 12-month-old bilingual infants showed similar left inferior frontal cortex activation to native and non-native consonant contrasts. On the other hand, monolingual (English) infants of the same age showed left inferior frontal cortex activation in response to native but not non-native contrasts. This study has been interpreted as evidence for the claim that bilingual infants can discriminate non-native phonetic contrasts until a later age than their monolingual counterparts (Berken et al., 2017; Birdsong, 2018; Burnham et al., 2017; Costa & Sebastián-Gallés, 2014; Jasińska & Petitto, 2014; Kandhadai et al., 2014; Kovelman et al., 2015; Liu & Kager, 2018; Potter & Saffran, 2015; Zadina, 2015). However, as Petitto et al. (2012) do not report a behavioural measure of perceptual discrimination, it remains unknown whether bilingual infants show a behavioural difference in their perceptual responses to non-native phonetic contrasts until a later age than monolinguals.

Mixed behavioural evidence exists for an effect of bilingualism on non-native contrast perception. For example, Liu and Kager (2015) tested sensitivity to a non-native aspiration consonant contrast (“/p/” — “/p^h^/”) in eight- to nine-, 11- to 12-, and 14- to 15-month-old infants either learning Dutch (monolingual) or Dutch and French or Spanish (bilinguals). The consonant contrast was non-native for all monolingual and bilingual infants. The results showed that, at eight to nine months, bilinguals but not monolinguals discriminated the non-native consonant contrast. There were no group differences in discrimination in 11- to 12- or 14- to 15-month--old monolinguals and bilinguals. On the other hand, Singh et al. (2017) tested 10- to 11.5-month-old monolingual (English) and bilingual (English and Mandarin) infants’ looking behaviour to native and non-native voiceless Hindi dental-retroflex contrasts ("/ta/" – "/Ta/"). Using a "switch" variant of a visual habituation paradigm (Narayan et al., 2010), Singh et al. (2017) habituated infants to a language sound (in this case, "/ta/") before playing the same language sound ("same" condition) and a contrastive sound ("switch" condition). This facilitates a comparison of monolingual and bilingual infants’ novelty responses to a non-native phonetic contrast (Narayan et al., 2010). Within-group analysis of the bilinguals revealed a significant increase in fixation time to the novel, contrastive non-native stimulus that was not present in the same analysis of the monolingual group. These results suggest that 10- to 11.5-month--old bilingual, but not monolingual, infants may be able to perceive non-native phonetic contrasts. However, the groups were not statistically compared, and thus group differences cannot be claimed from this study alone (Makin & Xivry, 2019).

Further evidence was presented in an unpublished thesis by Casaus (2015), who conducted a cross-sectional study of monolingual (either Spanish or Catalan) and bilingual (both Spanish and Catalan) children’s responses to non-native (Hindi) phonetic contrasts at seven-, 12-, 15-, and 18-month-old time points. Using the “switch” variant of a visual habituation paradigm as per Narayan et al. (2010) and Singh et al. (2017), the infants were habituated to a Hindi dental "/ta/" syllable whilst black-and-white checkerboards were presented. Once an infant habituated, indicated by a 40 % decrement in looking time to screen, a series of either dental "/ta/" syllables or retroflex "/Ta/" syllables were presented (Narayan et al., 2010). At 12 months, there was a non-significant trend (*p* = .055) of a *Group* x *Trial* interaction that predicted the outcome of looking time. Post-hoc results revealed that monolinguals did not look longer to the non-habituated (retroflex “/Ta/”) than the habituated (rental “/ta/”) sound, whilst bilinguals did, indicating potential non-native contrast discrimination in the bilingual but not monolingual group. This finding broadly aligns with previous research reporting bilingual discrimination of a non-native contrast at 12 months (Singh et al., 2017). At 15 months, results revealed a statistically significant *Group* x *Trial* interaction (Casaus, 2015). Post-hoc analyses suggested that the interaction was driven by the bilinguals looking longer at the non-habituated (retroflex “/Ta/”) compared to the habituated sound (dental "/ta/"), whilst no difference was observed in monolinguals. This suggests that at 15 months, bilinguals can discriminate the “/ta/” – “/Ta/” contrast, whilst monolinguals may not. Importantly, at 18 months, there was no indication in a within-group analysis that bilingual infants could discriminate the non-native contrast (Casaus, 2015). Overall, these findings suggest there is no evidence of monolinguals’ discrimination of a non-native consonant contrast at 12, 15, or 18 months, whilst there is evidence of discrimination in bilinguals at 12 and 15 but not 18 months.

These unpublished findings may indicate a behavioural effect of a prolonged period of sensitivity to a non-native retroflex consonant contrast amongst bilingual infants, though several key questions remain. Some research reports bilingual effects on perceptual sensitivity for non-native language sounds in the beginning of the second year of life (e.g., Singh et al., 2017), whilst others do not (e.g., Kalashnikova et al., 2023; Liu & Kager, 2015). The specific developmental trajectory of infants’ potential sensitivity to non-native contrasts within the 15- to 18-month age window also remains unclear, given that previous research has primarily been cross-sectional (Kraemer et al., 2000). It is still unknown whether a potential group difference is linked to a bilingual experience more broadly, or if it is specific to bilinguals learning language pairs that are linguistically similar (such as Spanish-Catalan bilinguals). The present study addresses these questions.

### 1.1 Hypotheses

It was predicted that simultaneous bilingual infants exposed from birth to English and another language without a retroflex consonant would retain perceptual sensitivity for non-native phonetic contrasts longer than would monolingual infants. The stimuli consisted of a non-native consonant contrast (Hindi dental "/ta/" versus retroflex "/Ta/") used in past perceptual narrowing research to report group differences between bilinguals and monolinguals in the first and second years of life (Casaus, 2015; Petitto et al., 2012; Singh et al., 2017; Werker & Tees, 1984; Werker et al., 1981). Eye-tracking was used to measure looking time differences to habituated (dental "/ta/") and novel (retroflex "/Ta/") tokens.

It was further predicted that the Hindi contrast would be imperceptible to all English monolingual infants tested, regardless of age (15 to 18 months) (Werker & Tees, 1984). These infants were expected to look at both trial types (habituated same versus novel switch) of the Hindi contrast equally. In contrast, a prolonged window of sensitivity to non-native contrasts for the bilingual group was anticipated, reflected by different looking patterns to habituated same versus novel switch tokens. A gradual decrease in bilingual infants’ sensitivity to non-native phonetic contrasts across the age range of 15 to 18 months was expected.

The study design, which allowed for age to be examined as a continuous variable, permitted a direct test of whether monolinguals and bilinguals demonstrated a difference in slope between the 15- and 18-month-old time points. This approach builds on previous research, most of which is cross-sectional, providing the opportunity to make direct inferences about developmental trajectories (Kraemer et al., 2000). A linear mixed effects model was constructed to test whether the looking time to the test phase was predicted by the interaction of *Group* x *Trial* x *Age*. A significant interaction was hypothesised, driven by bilingual infants’ prolonged sensitivity (compared to the monolingual group) to non-native consonant contrasts that was expected to decline with age.

### 1.2 Exploratory hypotheses

As reviewed above, bilingualism is a widely varying experience. If it is indeed the case that experience with two native languages drives a period of protracted sensitivity to non-native phonetic contrasts, it is logical to predict that infants who experience their two languages more frequently (e.g., 50 % Spanish, 50 % English), would retain this sensitivity longer than infants whose exposure to two languages is less balanced (e.g., 20 % Spanish, 80 % English). A “degree of bilingualism” metric, used in previous developmental research (Incera & McLennan, 2018; Nguyen et al., 2023; Mousley et al., 2022), can be calculated from in-depth language background interviews with infants’ parents. The impact of degree of bilingualism on bilinguals’ declining perceptual sensitivity to non-native contrasts remains an open question.

If their parents are bilingual, bilingual infants are also exposed to different habits in “language mixing”, a behaviour defined as switching languages mid-sentence or borrowing words from one language when using the other (see for example Byers-Heinlein et al., 2020; Tsui et al., 2020). It could be that bilingual children who are exposed to more language mixing at home, and therefore more unpredictable or variable speech streams, remain open to non-native contrasts later into life than do monolinguals.

### 1.3 Methodological aims

Previous perceptual narrowing research has relied on online computer mouse or key presses by the experimenter to calculate habituation and record infant looking times (for example, see Estes et al., 2007; Fennell & Waxman, 2010; Graf Estes & Bowen, 2013; Oakes et al., 2019; Polka et al., 2014; Singh et al., 2017; Sundara et al., 2008; for review, see Oakes, 2010). There are inherent disadvantages of button-press measurements for infant looking time. Collecting data with infants often requires monitoring many things at once, which can make it challenging for the experimenter to capture looking time measurement precisely, especially if they are working alone. Speech perception task trials also tend to be short, which could make the impact of even small imprecisions in button-press measurement proportionately more significant than tasks with longer trials. It is often the case that experimenters cannot be blinded to the conditions of perceptual narrowing paradigms, such as visual habituation paradigms, where the experimenter knows the design of the task and, from meeting the family, if the child is monolingual or bilingual. To address these issues and improve overall precision of looking time measurement, the habituation software PyHab (Kominsky, 2019) was modified to accept Tobii TX300 eye-tracking input to serve as the primary coder. Eye-tracking is temporally precise, capturing blinks and rapid saccades away from and back to the screen that cannot be captured by experimenter button presses. Because this software integration was novel, offline video recordings were used to validate eye-tracking looking time measurements. Manual, frame-by-frame measurements are more robust to large movements than eye-tracking, which can offer benefit as it is common for children participating in eye-tracking studies to lean forwards, backwards, or make other big movements during the task (Tomalski & Malinowska-Korczak, 2020). More detail about this software integration and its validity is included below.

## 2 Method

### 2.1 Power analysis

To our knowledge, no published analyses have yet reported the estimated effect size of the potential bilingual effect on non-native consonant contrast sensitivity in the second year of life. The necessary sample size to address this question was calculated using unpublished data from a cross-sectional study of non-native phonetic perception amongst simultaneous bilinguals at seven, 10, 15, and 18 months of age (Casaus, 2015) using a visual habituation paradigm.

At 15 months, Casaus (2015) reported a significant interaction of *Group* (monolingual versus bilingual) x *Trial* (same versus switch) (*F*(1, 42) = 6.27, *p* = .02). At 18 months, a within-participants ANOVA (bilinguals only) showed a null effect of test trial type (same versus switch) on looking time (*F*(1, 19) = 0.36, *p* = .55). The proposed analyses were designed to address whether or not monolinguals and bilinguals demonstrated a difference in slope between the 15- and 18-month-old time points, which were tested cross-sectionally in Casaus (2015). Casaus (2015)’s interaction statistic at 15 months was used to calculate the effect size at the point of maximal effect. At 15 months, Casaus (2015) reported a significant *Group* x *Trial* interaction (15 months, *F*(1, 42) = 6.27, *p* = .02) driven by increased sensitivity to the non-native contrast in the bilingual compared to the monolingual group. As recommended by Lakens (2013), the partial eta-squared value was calculated using the following formula: $\eta_{p}^{2}=\frac{F*df_{effect\;}}{\left(F*df_{effect\;}\right)+df_{error\;}}$ (in this case: $\eta_{\text{p}}^{2}=\frac{6.21*1}{(6.27*1)+42}=0.13$). Using Cohen’s (2013) equation for effect size of $f\left(f=\frac{\surd \eta_{p}^{2}}{1-\eta_{p}^{2}}\right)$, an effect size of *f* = 0.39 was calculated. A simulation-based power calculation for repeated measures, mixed effects ANOVA (within-between interaction) was conducted using the protocol laid out by Lakens and Caldwell (2021). Conducting 1000 simulations using parameter estimations from Casaus (2015), the analysis revealed that a sample size of *n* = 37 was required to detect the effect with 91.20 % power. Considering approximately 25 % attrition for an infant eye-tracking study (Althaus & Marechal, 2012; Mercure et al., 2018; Pons et al., 2015), it was expected that approximately *n* = 48 infants per group (*N* = 96) would need to be tested.

### 2.2 Participants

Data from a total of *N* = 95, 15 to 18 month olds was collected at the Birkbeck Babylab in central London. For failure to complete the task, *n* = 13 infants were excluded (*n* = 6 monolinguals and *n* = 7 bilinguals). For failure to reach habituation criteria within the window of between nine and 33 trials, *n* = 9 infants were excluded (*n* = 4 monolinguals, *n* = 5 bilinguals). For failure to look to the same or switch phases for at least one second each, a further 11 infants were excluded (*n* = 5 monolinguals, *n* = 6 bilinguals). This left a total of a *N* = 62 infants who contributed data to analysis. This total sample size fell short of the target by *n* = 12 participants (*n* = 5 bilingual) because of the COVID-19 pandemic that was ongoing when the funding supporting this project ended (further detail about implications found in discussion Section 4.1).

Of participants included in the analyses, *n* = 30 were monolinguals learning English only (*n* = 17 female, *M* = 516.83 days or 16.99 months, *SD* = 32.77 days or 1.08 months, *range* = 459-577 days or 15.09 to 18.97 months). The *n* = 32 bilinguals were learning English and another language that did not contain a retroflex consonant (*n* = 16 female, *M* = 518.31 days or 17.04 months, *SD* = 33.36 days or 1.10 months, *range* = 460-573 days or 15.12 to 18.84 months). Bilinguals’ non-English languages were Cantonese (*n* = 2), Czech (*n* = 1), Danish (*n* = 2), Dutch (*n* = 2), French (*n* = 2), Greek (*n* = 1), Hebrew (*n* = 1), Hungarian (*n* = 1), Indonesian (*n* = 1), Italian (*n* = 3), Mandarin (*n* = 2), Polish (*n* = 2), Russian (*n* = 2), Spanish (*n* = 6), Swedish (*n* = 1), Twi (*n* = 1), Welsh (*n* = 1), and Yoruba (*n* = 1). There were no differences in the groups’ ages (*t*(59.86) = -0.18, *p* = .861), and average household income was matched across groups. Mothers’ and fathers’ median level of education was a degree/higher national diploma in both groups. Ethical approval was granted both by University College London (4966/003) and Birkbeck, University of London (181960) ethics committees.

To ensure no bilinguals had experience with the non-native retroflex contrast, *Phoible*, an online repository of cross-linguistic phonological inventory data from 2186 distinct languages (Morgan & McCloy, 2019) was used. Ten retroflex consonants that contain more than 0 % "Representation" in the database were selected and a searchable table of 1767 exclusion languages was created. Infants with experience of these languages were excluded in the recruitment phase. Infants with more than 5 % exposure to third/-fourth languages were also excluded.

### 2.3 Bilinguals’ early language experiences

#### 2.3.1 Language exposure questionnaire

Percentage of language exposure was calculated using the English adaptation of the Language Exposure Questionnaire (“LEQ”) designed by Bosch and Sebastián-Gallés (1997) and often implemented in developmental research on bilingualism (Carbajal & Peperkamp, 2020; DeAnda et al., 2016; Kalashnikova et al., 2020; Mousley et al., 2022; Potter et al., 2018; Ramon-Casas et al., 2009; Singh & Tan, 2021). The interview took approximately 15 minutes, during which parents were asked about a typical day in the child’s life for each day of the week across different periods of time since birth.

The number of hours a child hears their native languages was calculated, a percentage score was produced, and the inclusion criteria of a minimum of 20 % exposure to the minority language and 80 % maximum exposure to the majority language was checked. A measure of “degree of bilingualism” was calculated as the percentage of exposure to the less dominant language divided by percentage of exposure to the more dominant language. For example, a child with 30 % exposure to English and 70 % exposure to Spanish would have a degree of bilingualism of 0.43. A score nearer to 1.0 indicates a 50/50 split in language exposure, whilst a score nearer to 0.25 indicates a 20/80 split between native languages. The bilingual sample, on average, reported a degree of bilingualism of *M* = 0.43 (*SD* = 0.19, *range* = 0.25-0.75). Bilinguals’ average percentage of exposure to English was *M* = 61.47 % (*SD* = 21.03 %, *range* = 20-80 %). Bilinguals’ average percentage of exposure to a non-English language was *M* = 38.53 % (*SD* = 21.03 %, *range* = 20-80 %). The distribution of bilinguals’ degree of bilingualism was normal (*W* = 0.97, *p* = .413) and evenly spread between the criteria of 20 % minority language minimum and 80 % majority language maximum.

#### 2.3.2 Language mixing scale

Each bilingual parent also completed a language mixing scale (Byers-Heinlein, 2013; Byers-Heinlein et al., 2020; Tsui et al., 2020) that took approximately three to five minutes. Parents were asked to rate on a scale of zero (Never) to six (Always) how often they mixed their language use. The questions asked about behaviours like borrowing words from another language or code switching between two languages in the same sentence. Bilingual parents reported, on average, a language mixing score of *M* = 13.28 (*SD* = 7.48, *range* = 0-30) out of a total possible 36 points on the scale. The distribution of bilingual infants’ parents’ language mixing scores was normal (*W* = 0.97, *p* = .420).

### 2.4 Eye-tracking procedure

The eye-tracking task was the first of a large battery to test the effects of bilingualism on emergent communication patterns. The total protocol took approximately 1.5 to 2.5 hours from start to finish. The protocol began with a 10- to-15-minute eye-tracking session that began with the non-native speech perception task reported here. Children sat on their parent’s laps approximately 65 centimetres away from a Tobii Pro TX300 eye-tracker in a dimly lit, featureless room. The eye-tracker was set to a sampling rate of 120 Hz (measurement accuracy: 0.4° screen size: 58.42 centimetres; aspect ratio: 16:9; screen resolution: 1920 × 1080). The tracking equipment and stimulus presentation were controlled on a Dell 2018 desktop computer running Windows 10 via PsychoPy3 (Peirce et al., 2019) and PyHab, a programme designed specifically for infant looking time measurements during habituation paradigms (Kominsky, 2019). A camera mounted directly above the horizontal midpoint of the screen recorded the child’s behaviour.

### 2.5 Experimental paradigm

A classic switch variant of an infant visual habituation paradigm was used to compare monolingual and bilingual infants’ novelty responses to a non-native, Hindi dental-retroflex “/ta/” – “/Ta/” phonetic contrast (Narayan et al., 2010). This design has been implemented widely in previous research, allowing for the comparison of monolingual and bilingual infants’ perceptual sensitivities to phonetic contrasts in a non-native language (for example, see Narayan et al., 2010; Singh et al., 2017).

**Fig. 1:**
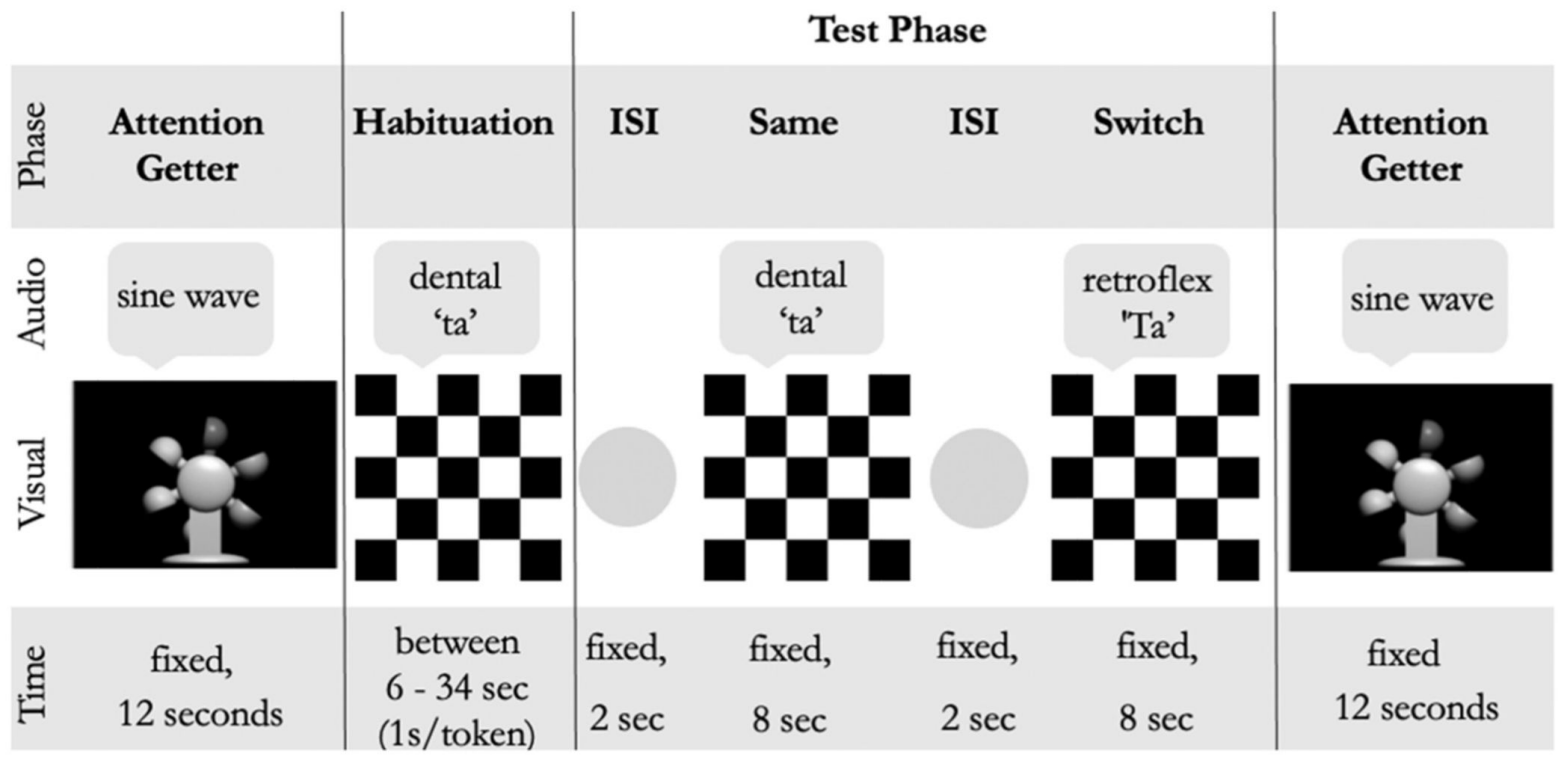
Depiction of experimental paradigm. Note. ISI = interstimulus interval. 1 s/token = one second per token shown in the habituation phase, broken into three "windows" of three trials each. Number of tokens in the habituation phase were variable (between six and 34) and depended on when the infant looked to the screen 40 % less than their longest looking time measurement to a previous window.

Auditory stimuli were presented through loudspeakers located behind the computer screen. A static picture of a black and white checkerboard was presented on a screen concurrently with auditory stimuli. These stimuli have been used previously with a variety of different speakers and are described in detail elsewhere (e.g., duration, average pitch, pitch minimum and maximum, pitch range, etc.) (Casaus, 2015; Petitto et al., 2012; Werker & Tees, 1984; Werker et al., 1981). The task began with a non-social attention-getter (i.e., a whirling water wheel, see Fig. 1) at the centre of the screen accompanied by an auditory tone sine wave for a fixed duration of 12 seconds (following Casaus, 2015). This allowed for a baseline measure of infants’ attention to the sequence at the beginning and end of the task. The habituation phase followed the first attention-getter (see Fig. 1). During the habituation phase, the programme calculated and stored the child’s peak looking time, calculated as the longest amount of time the child looked towards three consecutive tokens of the habituation phase (Oakes, 2010). After every set of three tokens, starting with the sixth token, the child’s mean looking time (over three tokens) was compared to the peak looking time. If the child’s looking time to the most recent window of three tokens was equal to or less than 60 % of the child’s peak looking time (Narayan et al., 2010), the child was considered habituated. At this point, the programme ended habituation and proceeded with the presentation of the inter-stimulus interval, which was a two-second flashing yellow light (see Fig. 1).

Next was the test phase, which contained two conditions: habituated same (dental "/ta/") and novel switch (retroflex "/Ta/"). In these conditions, infants were presented with short audio clips repeatedly articulating the same dental "/ta/" in the same phase and the retroflex "/Ta/" in the switch phase (the same stimuli used by Casaus, 2015). The retroflex switch sound is a phonemic contrast to the habituated same sound, but non-native speakers of Hindi do not perceive the two tokens as different from each other (Werker & Tees, 1984; Werker et al., 1981). If infants were able to perceive the non-native contrast, it was hypothesised they would show a novelty response to the switch phase indicated by longer looking times to the screen (Narayan et al., 2010). If they could not perceive the contrast, they were expected not to show a novelty response and instead look for a short amount of time to the switch phase which, to them, sounded perceptually identical to the habituation phase. Within both conditions of the test phase, eight, one-second natural syllables were presented whilst a concurrent black-and-white checkerboard was shown on the screen (see Fig. 1). Each test phase (same and switch) had eight variations with diverse pitch and a variety of child-directed intonations. These were presented in random order (Oakes, 2010). The phases were separated by a flashing yellow inter-stimulus interval to regain infants’ attention.

### 2.6 Looking time measurement

Infant looking time was measured in two ways. A Tobii a TX300 remote eye-tracker was programmed to serve as the primary coder using Python 3.6.9 and the GitHub package called *psychopy_tobii_infant* (https://github.com/yh-luo/psychopy_tobii_infant). To check reliability of this measurement, looking time was also measured from video recordings of the protocol via offline, frame-by-frame coding (one frame = 40 ms, look/no-look).

### 2.7 Eye-tracking measurement reliability

**Fig. 2:**
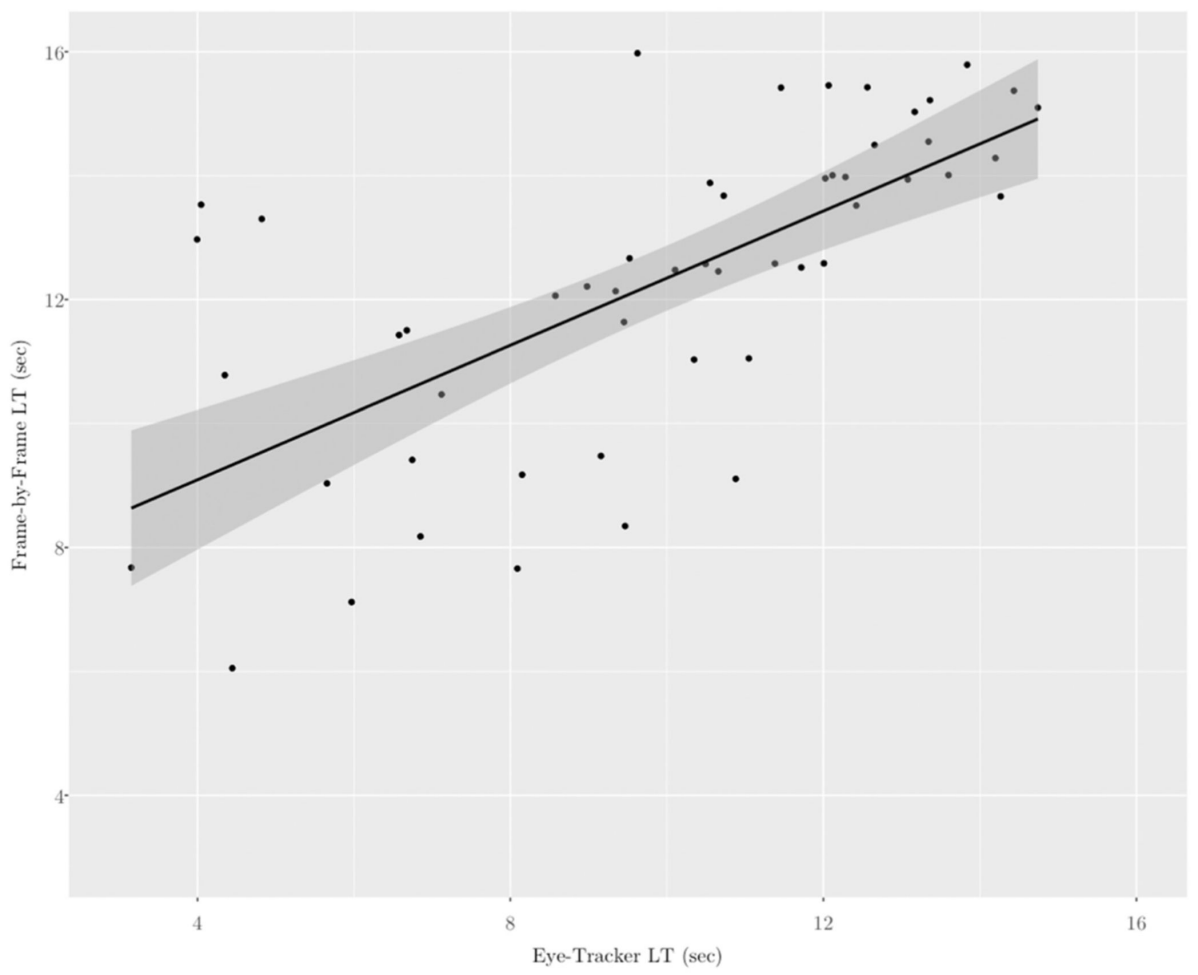
Reliability of looking time measures. Note. LT = Looking time. Shaded grey = standard error.

Frame-by-frame video coding was conducted in ELAN (ELAN, 2019). Using the frame-by-frame function (frame = 40 milliseconds), each child’s gaze was binary coded as LOOK or NO-LOOK to the screen. When the child moved temporarily out of the webcam view, the gaze was coded as NO-LOOK to be conservative. Each child’s total looking time to the screen, calculated as the sum number of seconds of looking in the LOOK tier, was extracted. The result was two measures of looking time for each phase of the task, one calculated online by the eye-tracker and one calculated offline by frame-by-frame coding. There was a strong correlation between the two looking time measures in the test phase, indicating that the measures were highly reliable (*r* = 0.71, *p* < .001, Fig. 2).

## 3 Results

### 3.1 Main analysis

The main hypothesis was tested with a linear mixed effects model using lme4 (Bates et al., 2015) in R (R Core Team, 2021) and the lmerTest package (Kuznetsova et al., 2017). Assumptions of linear mixed effects models were met. The predictors included in the linear mixed effects model were *Group* (monolingual versus bilingual), *Trial* (same versus switch), and *Age* (continuous, days), and the outcome was looking time in seconds (full model: Looking Time ~ *Group* x *Trial* x *Age* + (1|ID)). The hypothesised interaction of *Group* x *Trial* x *Age* was not significant (*p* = .737). There were no main effects of group (*p* = .673), trial (*p* = .814), or age (*p* = .396). Finally, the step() function of lmerTest was used to conduct backwards elimination of fixed effects terms in the linear mixed effects model. The results revealed the best fitting model included only the random effect of individual.

### 3.2 Exploratory analyses

#### 3.2.1 Potential discriminators

**Fig. 3:**
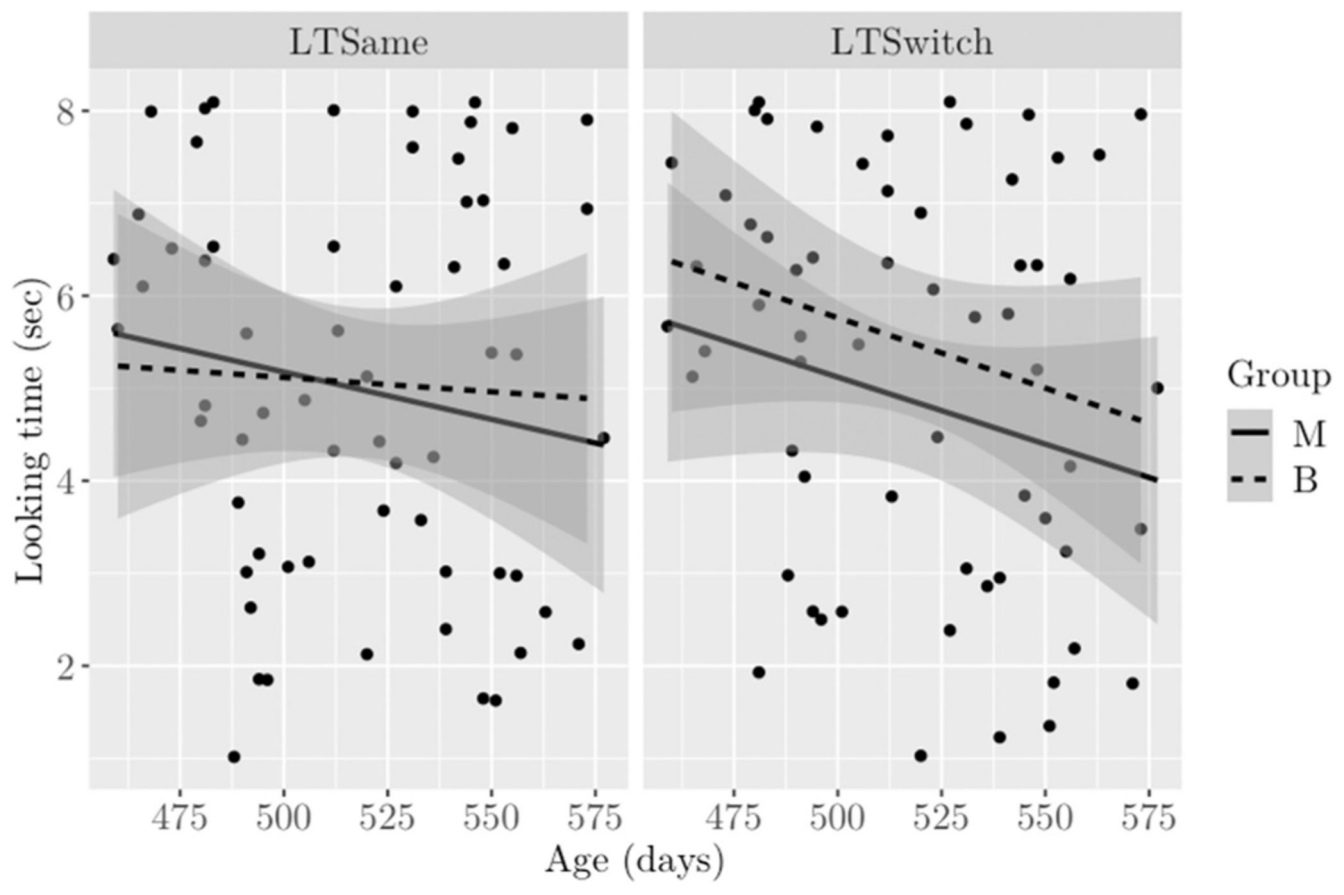
Infants’ looking time to test phase by age. Note. LTSame = same phase. LTSwitch = switch phase. Shaded area = standard error. Bilinguals represented by the dashed line and monolinguals by the solid line.

In alignment with previous research, the present study was conducted under the expectation of a novelty preference for the switch phase if infants discriminated the contrast. Under this premise, to measure whether any children were potentially discriminating the contrast, a “novelty preference score” was calculated as $\frac{\;LT\;Switch-LT\;Same\;}{\;LT\;Switch\;+\;LT\;Same\;}$. This identified potential discriminators whilst standardising for individual differences in overall looking time to the test phase (see Fig. 3). Children who looked longer to the switch phase than to the same phase would have a novelty preference score of more than zero. Children who looked either to the phases equally or to the same phase more than to the switch phase would have a novelty preference score of equal to or less than zero.

**Fig. 4:**
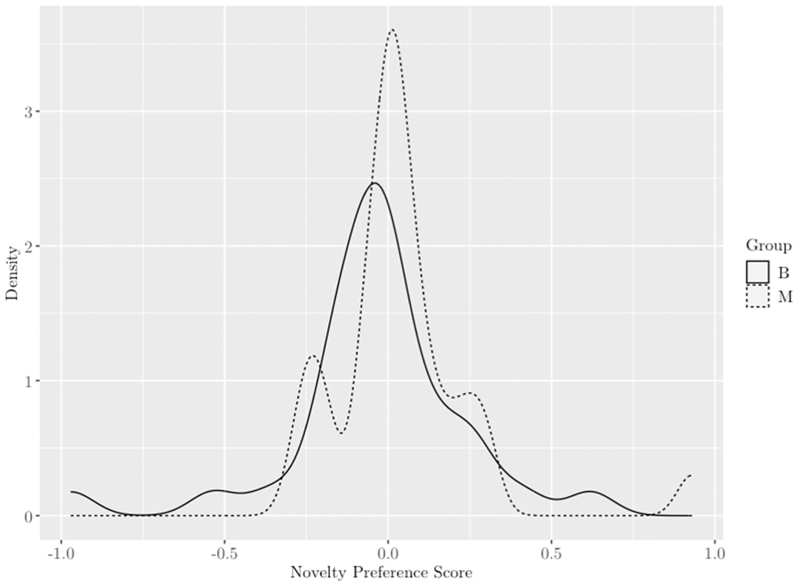
Distributions of novelty preference scores by group. Note. Novelty preference score calculated as looking time to switch phase minus looking time to same phase divided by sum of total looking to same plus switch. Distribution of bilinguals represented by solid line and monolinguals by dashed line.

A total of 31 (out of 62) children had a novelty preference score of more than zero, suggesting they may have been discriminating the non-native phonetic contrast. Of them, nine were 15 month olds (*n* = 6 monolinguals, *n* = 3 bilinguals), three were 16 month olds (*n* = 2 monolingual, *n* = 1 bilingual), 11 were 17 month olds (*n* = 6 monolinguals, *n* = 5 bilinguals), and eight were 18 month olds (*n* = 4 monolingual, *n* = 4 bilingual) (total monolingual: *n* = 18, total bilingual: *n* = 13) (see Fig. 4).

#### 3.2.2 Group differences in habituation

**Fig. 5:**
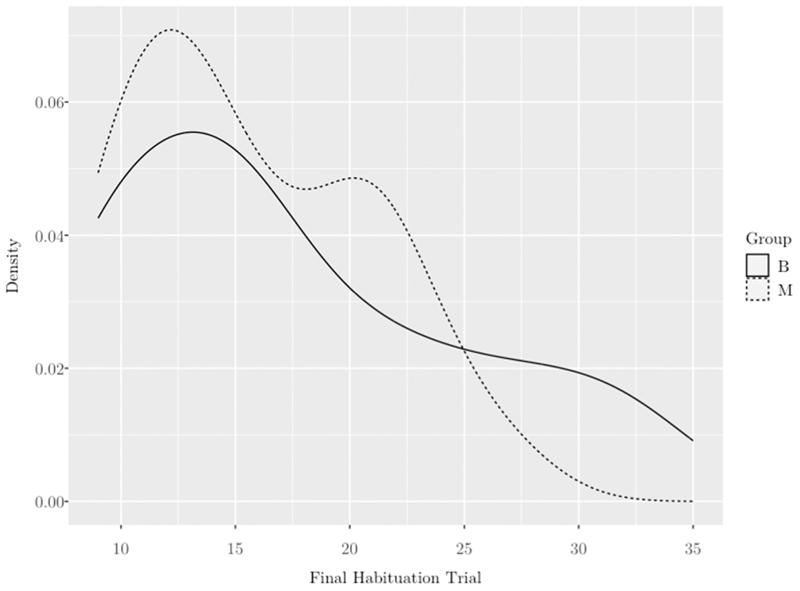
Distributions of habituation thresholds by group. Note. Final habituation trial = the point at which infants reached the 40 % decrement in looking time and task moved to the test phase. Bilinguals represented by solid line and monolinguals by dashed line.

There were no group differences in overall amount of looking to the habituation phase (*t*(55.80) = 0.71, *p* =. 483) and a Pearson’s chi-square revealed no differences in the groups’ average last habituation trial (*p* = .450) (see Fig. 5).

#### 3.2.3 Within-group variability

Within the bilingual group, there was no relationship between novelty preference score and degree of bilingualism (*t*(30) = 0.10, *p* = .328) nor between novelty preference score and amount of parental language mixing (*t*(30) = 1.71, *p* = .100). Of the *n* = 13 potential bilingual discriminators, degree of bilingualism ranged from 0.10 to 0.66 (*M* = 0.45, *SD* = 0.17), language mixing ranged from 4.5 to 29 (*M* = 14.69, *SD* = 7.25), and non-English languages included Cantonese (*n* = 2), Danish (*n* = 1), French (*n* = 1), Greek (*n* = 1), Hebrew (*n* = 1), Hungarian (*n* = 1), Italian (*n* = 2), and Mandarin (*n* = 2), and Spanish (*n* = 2).

## 4 Discussion

On the basis of neuroimaging data, it has been proposed that bilingual infants remain sensitive to foreign speech sounds later into life than do monolinguals (Petitto et al., 2012). The theory posits that the strong linguistic demands of spoken language bilingualism, including high phonetic variability and the need to navigate between two spoken languages, may drive a “perceptual wedge” in bilinguals’ declining sensitivity to non-native speech sounds (Petitto et al., 2012). While this notion is supported by some neurophysiological studies showing distinct neural responses in bilinguals towards the end of the first year of life, direct behavioural tests of this proposal are limited. The present study filled this gap by testing whether monolinguals and bilinguals differed in the trajectories of their declining sensitivity to a non-native phonetic contrast between 15 to 18 months. No significant group differences were found.

### 4.1 No evidence of group differences in non-native contrast sensitivity

The main hypothesis predicted that bilingual infants, who were exposed to English and a second language from birth, would retain perceptual sensitivity to a non-native phonetic contrast later into life than would monolinguals (e.g., per the “Perceptual Wedge” hypothesis, Petitto et al., 2012). Considering previous research that suggests monolinguals are no longer able to perceive foreign language sounds after 12 months of age (Best, 1994; Kuhl et al., 1992; Polka & Werker, 1994; Werker & Tees, 1984), it was expected that all English-learning monolinguals between 15 and 18 months would not be able to perceive the foreign contrast (Hindi retroflex “/ta/” and “/Ta/”, as per Werker & Tees, 1984). Bilingual infants, to whom the contrast was also foreign, were expected to show sensitivity to the non-native contrast in the younger months of the age window (e.g., 15, 16 months). This was expected to decline towards the end of the age window (i.e., 18 months).

The main analyses did not support this hypothesis. There were no indications that monolinguals or bilinguals reliably discriminated the foreign contrast at any age studied. While no criteria exist against which it can be conclusively determined if a child is discriminating a phonetic contrast, a predominant assumption made in developmental speech perception research is that children will look longer to new sounds than familiar and/or habituated ones (e.g., Albareda-Castellot et al., 2011; Bosch & Sebastián-Gallés, 2003; Kuhl et al., 2006; Narayan et al., 2010; Sebastián-Gallés & Bosch, 2009; Singh et al., 2017; Werker & Tees, 1984). In the present study, a novelty preference score was calculated for each child to determine if they did indeed look longer to the novel, contrastive “/Ta/” token after habituating to the dental “/ta/”. The novelty preference calculation did not reveal any group differences. Approximately half of all infants were identified as potential discriminators, and they were spread evenly across groups and ages (see Fig. 4).

The lack of group differences between monolinguals and bilinguals do not support the “Perceptual Wedge” hypothesis (Petitto et al., 2012). The findings also do not align with the pattern reported by Casaus (2015), who found 15-month-old bilinguals, but not monolinguals, discriminated a non-native consonant contrast. One possibility is that findings diverge because of differences in analysis. Casaus (2015) used a cross-sectional design which permitted *n =* 44 (*n =* 22 monolinguals) in the 15-month-old group. In contrast, the trajectory approach adopted here meant there was a smaller sample size at each month. Only 13, 15 month olds were included in the current study. If the perceptual narrowing effect is limited to the 15th month alone, it is likely that the present study did not have enough power within that limited age group to detect it. The strength of the approach is that it could capture variability in the developmental trajectories of children across the 15-to-18-month age window, but it is likely not sensitive enough to capture small effects at the level of a month-by-month comparison. Future research may consider testing large groups of younger infants to determine, with enough power to test group equivalence (Lakens et al., 2018), if group differences shown previously are driven predominantly by younger ages than studied here.

Importantly, the sample collected here was *n =* 12 children short of the target recruitment size due to ongoing COVID-19 lab closures while the funding supporting this work ended. However, if it were simply the case that the present study was unpowered, there would likely be a higher number of potential bilingual discriminators of the contrast in the 15th and 16th month, even if the difference were non-significant, which is not reported. If data from the last *n =* 5 bilingual and *n =* 7 monolingual children had been collected, and if all children showed strong effects that aligned with the hypothesis such that all bilinguals showed a novelty preference and all monolinguals did not, the final result would be exact group equivalence (*n* = 18 potential discriminators in each group). For this reason, it is unlikely that the addition of these last few children would significantly change this study’s results or the interpretation of them.

Although it is thought to be a universal phenomenon, some perceptual narrowing research has failed to show evidence of phonetic contrast discrimination in infants that aligns with typically proposed perceptual narrowing trajectories (e.g., Mazuka et al., 2013; Tyler et al., 2014; for review, see Singh et al., 2022). Some evidence suggests that bilinguals may construct a single language system in which they store and access information in both of their native languages (for review, see De Houwer, 1995). The notion of one cognitive model for language, even in the case of bilinguals’ two acquired language systems, could explain why monolinguals and bilinguals show similarities at certain ages and/or on certain tasks (for review, see Werker et al., 2009), such as in the domains of lexical development (Pearson et al., 1993, 1995) and speech comprehension (Shook & Marian, 2012). It could be that an alignment of canonical perceptual narrowing trajectories in monolinguals and bilinguals at certain points in development can also be explained by the theory of one cognitive system that accommodates two language systems. Future work is required to provide direct evidence, as the null results presented here cannot be interpreted as support of the null hypothesis (i.e., group equivalence). Future research may test whether similarities exist between monolingual and bilingual infants’ perception of non-native consonant contrasts by establishing statistical group equivalence (Lakens et al., 2018).

We could also speculate that potential bilingual effects on non-native speech perception follow a U-shaped trajectory that cannot be captured in the limited 15- to 18-month age window studied here. A non-linear trajectory in non-native contrast sensitivity would be characterised by initial discrimination from birth to approximately seven months of age, followed by a decrease in sensitivity between eight to nine months thought to accompany native language specialisation, and then later re-emergence of discrimination abilities. As described in the introduction, such U-shaped patterns have been shown in both monolingual and bilingual infants’ sensitivity to native and non-native tonal contrasts over the first two years of life (Liu & Kager, 2014, 2016). For example, Liu and Kager (2014) found that Dutch-learning monolinguals showed sensitivity to a non-native tonal contrast at five to six months of age and at 17 to 18 months, but not at nine months. Liu and Kager (2016) report a similar U-shaped trajectory in bilinguals learning Dutch and another non-tonal language, such that they showed evidence of non-native tonal contrast discrimination at five to six months and 11 to 12 months, but not at eight to nine months. In this context, experience-related effects in infants’ non-native speech sound discrimination may be understood as differences in the timing or nature of discriminatory re-emergence in bilinguals compared to monolinguals. The bilinguals in Liu and Kager (2016) showed evidence of sensitivity re-emergence about six months earlier (11 to 12 months of age) than the monolinguals in Liu and Kager (2014) (17 to 18 months). The approach of the present study – to investigate differences between 15- to 18-month-old monolingual and bilingual infants – is not well-suited to capture such broad, U-shaped effects that occur over the first years of life. Future large-scale, collaborative approaches (e.g., the *ManyBabies* project; Baumgartner et al., 2023; Frank et al., 2017) may be most effective in investigating infants’ declining and possibly re-emerging sensitivities to different types of native and non-native language sounds on a larger developmental scale (e.g., Kalashnikova et al., 2023).

It is important to note that the U-shaped bilingual effects reported in previous perceptual narrowing literature may be sensitive to both the sound inventories of bilinguals’ two languages (e.g., tonal versus non-tonal) and to the types of stimuli used. Kalashnikova et al. (2023) found no group differences in sensitivity to non-native Cantonese tonal contrasts between five-, 10-, and 17-month-old monolinguals (learning non-tone or pitch-accent languages) and bilinguals learning either two non-tone languages (Basque and Spanish) or one tonal and one non-tonal language (Mandarin and English). While the lack of robust differences between 17-month-old monolingual and bilingual infants in Kalashnikova et al. (2023) roughly aligns with the lack of group differences reported here, direct comparisons are difficult to draw. Kalashnikova et al. (2023) tested sensitivity to non-native tonal contrasts in bilinguals learning one tonal and one non-tonal language, whereas the current study tested non-native retroflex consonant contrast discrimination in bilinguals learning two languages without a retroflex consonant. Kalashnikova et al. (2023) also found that all monolingual and bilingual infants across the three time points discriminated the tonal contrast, whereas the results of this study did not suggest all infants discriminated the non-native retroflex consonant contrast.

Perhaps more conceptually similar to this study are the stimuli used by Liu and Kager (2015), who tested sensitivity to a non-native aspiration consonant contrast (“/p/” — “/p^h^/”) in eight- to nine-, 11- to 12-, and 14- to 15-month-old infants either learning Dutch (monolingual) or Dutch and French or Spanish (bilinguals). Liu and Kager (2015)‘s results showed that, at eight- to nine-months, bilinguals but not monolinguals discriminated the non-native consonant contrast. There was no evidence of discrimination in either group at 11- to 12-month and 14- to 15-month timepoints (Liu & Kager, 2015). Lack of non-native contrast discrimination in the second year of life, in both monolingual and bilingual infants (Liu & Kager, 2015), broadly aligns with the lack of discrimination amongst 15- to 18-month-old monolinguals and bilinguals reported here. The current study found no evidence of reliable differences between monolingual and bilingual 15 to 18 month olds’ looking behaviours that would indicate presence of the predicted group differences on non-native phonetic contrast discrimination. Overall, future research is required to extricate fully the experience-related effects that diverse groups of infants show in their trajectories of sensitivity to different types (e.g., tonal and non-tonal) of non-native language sounds.

Another possible reason for the lack of coherence between the present study and previous studies in this area is that any difference in non-native contrast discrimination between monolinguals and bilinguals may be present at a younger developmental age than that studied here. The 15- to 18-month age window investigated was older than previous samples in which neurophysiological group differences between monolinguals and bilinguals have been suggested (Petitto et al., 2012; Singh et al., 2017). One study reported that 10- to 11.5-month-old bilinguals looked longer to the switch than same phase of a non-native phonetic contrast (Singh et al., 2017). This effect was not present in monolinguals (Singh et al., 2017). Further, Casaus (2015) reported a trend of a *Group* x *Trial* interaction effect, driven by a significant within-group bilingual discrimination effect at 12 months and a statistically significant *Group* x *Trial* interaction at 15 months. The 15- to 18-month-old age range was selected in the current study to test whether behavioural differences persisted past the point of neural differences reported by the four- to 12-month-old literature (Petitto et al., 2012; Singh et al., 2017) and to replicate the behavioural differences reported in unpublished research at 15 to 18 months (Casaus, 2015). However, it may be that behavioural group differences in monolingual and bilingual children’s sensitivity to non-native phonetic contrasts are stronger at the end of the first year and beginning of the second year (between 12 and 15 months) than at later time points. Future work should employ a developmental trajectory approach with this age range to determine if group differences between 12- to 15-month-old monolingual and bilingual infants’ looking to non-native contrasts is replicable, and if so, what characterises the groups’ different trajectories.

### 4.2 No evidence of within-group bilingual variability effects

Within-group, differences in bilinguals’ early language experiences were not related to their sensitivity to foreign language sounds. “Degree of bilingualism”, calculated as percentage of exposure to minority divided by majority language, and exposure to parents’ language mixing behaviours were not related to bilinguals’ contrast discrimination. When novelty preference was calculated for individual children, approximately half of all participants across the bilingual (and monolingual) groups showed a novelty preference that may have been linked to contrast discrimination. However, amongst potential bilingual discriminators, there were no clear patterns related to age, types of non-English languages (i.e., close versus distant language pairs), children’s degree of bilingualism, nor parents’ language mixing behaviours. The lack of relationships between bilingual experiences and novelty preference is not particularly surprising given that most of the bilinguals (59.37 %) did not show evidence of discriminating the non-native contrast. It is important to test whether elements of variability in bilingual experiences relate to processes of interest when this variability is the theoretical motivation for predicting bilingual differences from monolinguals. Measuring types of within-group variability can help clarify the environmental conditions that may drive group differences. The shift away from treating bilingualism as a categorical variable (for example, see Byers-Heinlein, 2015; Kremin & Byers-Heinlein, 2021) is another important consideration in future perceptual narrowing literature.

Another difference between the present study and past work is the diverse composition of the bilingual group. To understand if being raised bilingual has robust, cascading effects on the development of speech perception, it is necessary to test effects across diverse groups of bilinguals learning a variety of languages. The experience of learning two languages that are in the same language family and are therefore quite similar to each other, such as Spanish and Catalan, is likely to differ from learning two languages of different families, such as Chinese and Arabic (Floccia et al., 2018). The two languages a bilingual infant hears can also be variable in domains such as speech sound inventories, transitional probabilities, use of lexical stress, rhythm, and word order. Most previous research on perceptual narrowing in infancy constrains the language pairings of bilingual groups. The results that do exist about the effects of bilingualism on perceptual narrowing are derived primarily from bilinguals learning phonetically similar languages that are predominantly romance and/or Germanic languages (for review, see Singh et al., 2022; for experimental examples, see Albareda-Castellot et al., 2011; Bosch & Sebastián-Gallés, 2003, 2005; Burns et al., 2007; Sebastián-Gallés & Bosch, 2009; Sundara et al., 2008).

One study reporting a bilingual effect on perceptual narrowing in 12- to 15-month-old children tested a sample of bilinguals learning Spanish and Catalan, two languages that are phonologically close to each other and contain similar translational probabilities, rhyme, and word order (Bosch & Sebastián-Gallés, 1997; Casaus, 2015). It may be that those learning two phonologically close languages lose the ability to perceive non-native contrasts later in life than those learning distant languages. In a study of bilinguals learning two phonologically distant languages (Mandarin and English), Singh et al. (2017) found a bilingualism effect in 10- to 11.5-month-old participants. It could be that a bilingual effect on perceptual narrowing is related to the distance between the two languages a bilingual is learning. Bilinguals learning two close languages that have a high degree of overlap with each other, such as Spanish and Catalan, may remain more sensitive to subtle differences in speech sounds than those whose native languages are distant and therefore rapidly identifiable based on large phonological differences, like Mandarin and English. Group differences between monolinguals and distant-language bilinguals may be best captured towards the end of the first year whilst close-language bilinguals may still differ from monolinguals towards the middle of the second year. This is an open question for future research.

The current study cannot empirically test whether language distance relates to trajectories of perceptual narrowing; however, it is relevant that the *n =* 13 potential bilingual discriminators were learning two languages with differing distances from each other. Some were learning English and non-English language pairs that were distant, such as Cantonese, Greek, Hebrew, Hungarian, and Mandarin, whilst others were learning languages more closely related to English such as Danish, French, Italian, and Spanish. It could be that the heterogeneity of this study’s bilingual sample masked differences that are language-pair specific. Future work should determine the time at which bilinguals learning two phonologically distant language pairs are no longer able to discriminate non-native consonant contrasts, as well as determine more specifically the time at which learners of two similar languages lose this perceptual skill. In a recent meta-analysis, Singh et al. (2022) surveyed 99 perceptual narrowing studies and reported that, of the 19 % that examined both monolingual and bilingual infants, half sampled learners of English, Catalan, and Spanish. The present study adds to the existing literature by investigating perceptual narrowing in a group of heterogenous bilinguals, but future research is clearly required to fully understand the developmental effects of bilingualism on foreign speech sound perception.

### 4.3 Methodological considerations

The eye-tracking integration approach developed for this study may be of interest to future researchers using a habituation protocol. Visual habituation paradigms often rely on experimenter button-press measurements that are likely subject to error and experimenter bias. The current study used an open source looking time and stimulus presentation system, PyHab (Kominsky, 2019), a PsychoPy (Peirce et al., 2019) add-on. Instead of relying on button-presses, the software was amended to accept input from a Tobii TX300 eye-tracker to serve as the primary gaze coder. Manual, frame-by-frame looking time measures were established to allow reliability checks of the eye-tracking approach. These analyses clearly demonstrated that the eye-tracking method reliably measured infants’ looking time during the habituation paradigm. Agreement between methods’ test phase measurements was high (*r =* 0.71). Unsurprisingly, disagreement between methods was generally higher in cases where children moved significantly during the session. There were four cases where measurement difference was greater than two standard deviations above the mean. In all of them, the child moved significantly down or back, and frame-by-frame analysis captured valid looking time measurement that eye-tracking did not. Given that this occurred in 4 % of the sample tested, there does not appear to be a high degree of risk for unnecessary data loss, though future research with this method may want to predict attrition accordingly. Instances where frame-by-frame coding can recover “lost” eye-tracking data can also be addressed post-hoc; however, data replacement is complicated by the fact that visual habituation is determined categorically and irreversibly by the online eye-tracker measurement.

Reliability of habituation decisions between measures were also examined. The habituation calculation was determined online by eye-tracking; if a child’s eye gaze was lost before or during habituation, the software would have advanced into the test phase incorrectly, and this decision cannot be reversed post-hoc. Habituation windows were located in the experimental time course by referencing habituation token length against the experiment start time. Frame-by-frame looking times were extracted from timestamps in ELAN output files and infants’ total looking to each habituation window was summed. The trial at which infants reached habituation, according to frame-by-frame measurement, was calculated as it was online by the eye-tracker (i.e., as the point at which infants’ overall looking to a habituation window fell to 60 % of the looking time of the longest three-trial window). In all but the four cases of measurement disagreement referenced above, the frame-by-frame habituation trial matched the eye-tracker habituation trial, suggesting high agreement between the measures’ threshold decisions. These four cases were excluded from final analysis for failure to look for at least one second to the same and switch phases, and thus mismeasurement in habituation did not confound the results.

Overall, the eye-tracking integration used in this study to measure infant looking behaviour provided valid measurement of infants’ looking time during visual habituation paradigms. The method was generally robust, capturing infants’ eyes on the screen, even during a habituation paradigm where infants became increasingly less interested in the stimuli. It also seems promising for research with younger, less mobile infants, as valid data was collected in the present sample of highly mobile children who were old enough to crawl and walk. Future research should employ sensitive techniques, such as the eye-tracking integration used here, to re-examine the proposed trajectories of monolinguals’ narrowing to foreign language sounds. Precision of looking time measurement has previously been shown to affect the detection of potential monolingual and bilingual group differences in discrimination to native language sounds (see Albareda-Castellot et al., 2011). It is possible that more sensitive approaches than previously used will reveal greater variability in infants’ perceptual narrowing trajectories than is currently thought. The stimulus presentation software is freely available (https://github.com/jfkominsky/PyHab; Kominsky, 2019) and now contains a feature that allows the experimenter to select eye-tracking.

## 5 Conclusion

This study addresses recent calls for diversification of perceptual narrowing research to include new methods and diverse samples, such as bilinguals with a wide variety of language pairs (Singh et al., 2022). The results do not support the widely popular claim that heterogenous bilinguals retain perceptual sensitivity to non-native contrasts for longer than monolinguals (Berken et al., 2017; Birdsong, 2018; Costa & Sebastián-Gallés, 2014; Jasińska & Petitto, 2014; Kovelman et al., 2015; Potter & Saffran, 2015; Singh et al., 2017; Singh & Tan, 2021; Zadina, 2015). Variability within bilinguals’ experiences also did not show significant relationships to bilinguals’ discrimination of foreign language sounds. Lack of obvious bilingual effects could be related to the age range in which it was measured, the wide variability in bilinguals’ non-English languages, or to the more sensitive eye-tracking measurement than typically employed by perceptual narrowing research. While there were no obvious group differences, approximately half of all 15 to 18 month olds showed more looking time to the novel stimulus than the habituated stimulus, which may indicate discrimination of the foreign language sound in both monolinguals and bilinguals. Finally, an adaptation of a free stimulus presentation software, PyHab, was developed and validated for precise and objective measurement of infant looking time during a visual habituation paradigm. The adaptation has been integrated with the software and is publicly available for use.

## Data Availability

Data will be made available upon request for participants whose parents provided consent for their child’s information to be shared with external researchers.

## References

[R1] Albareda-Castellot B, Pons F, Sebastián-Gallés N (2011). The acquisition of phonetic categories in bilingual infants: New data from an anticipatory eye movement paradigm. Development Science.

[R2] Althaus N, Marechal D (2012). Using saliency maps to separate competing processes in infant visual cognition. Child Development.

[R3] Bates D, Mächler M, Bolker B, Walker S (2015). Fitting linear mixed-effects models using lme4. Journal of Statistical Software.

[R4] Baumgartner HA, Alessandroni N, Byers-Heinlein K, Frank MC, Hamlin JK, Soderstrom M, Voelkel JG, Willer R, Yuen F, Coles NA (2023). How to build up big team science: A practical guide for large-scale collaborations. Royal Society of Open Science.

[R5] Berken JA, Gracco VL, Klein D (2017). Early bilingualism, language attainment, and brain development. Neuropsychologia.

[R6] Best CT, Goodman JC, Nusbaum HC (1994). The development of speech perception: The transition from speech sounds to spoken words.

[R7] Best CT, Goldstein LM, Nam H, Tyler MD (2016). Articulating what infants attune to in native speech. Ecological Psychology: A Publication of the International Society for Ecological Psychology.

[R8] Best CT, McRoberts GW (2003). Infant perception of non-native consonant contrasts that adults assimilate in different ways. Language and Speech.

[R9] Birdsong D (2018). Plasticity, variability, and age in second language acquisition and bilingualism. Frontiers in Psychology.

[R10] Bosch L, Ramon-Casas M (2014). First translation equivalents in bilingual toddlers’ expressive vocabulary: Does form similarity matter?. International Journal of Behavioural Development.

[R11] Bosch L, Sebastián-Gallés N (1997). Native-language recognition abilities in 4-month-old infants from monolingual and bilingual environments. Cognition.

[R12] Bosch L, Sebastián-Gallés N (2003). Simultaneous bilingualism and the perception of a language-specific vowel contrast in the first year of life. Language and Speech.

[R13] Bosch L, Sebastián-Gallés N, Cohen J, McAlister KT, Rolstad K, MacSwan J (2005). Developmental changes in the discrimination of vowel contrasts in bilingual infants.

[R14] Burnham D, Singh L, Mattock K, Woo PJ, Kalashnikova M (2017). Constraints on tone sensitivity in novel word learning by monolingual and bilingual infants: Tone properties are more influential than tone familiarity. Frontiers in Psychology.

[R15] Burns TC, Yoshida KA, Hill K, Werker JF (2007). The development of phonetic representation in bilingual and monolingual infants. Applied Psycholinguistics.

[R16] Byers-Heinlein K (2013). Parental language mixing: Its measurement and the relation of mixed input to young bilingual children’s vocabulary size. Bilingualism: Language and Cognition.

[R17] Byers-Heinlein K, Schweiter JW (2015). The Cambridge handbook of bilingual processing.

[R18] Byers-Heinlein K, Burns TC, Werker JF (2010). The roots of bilingualism in newborns. Psychological Science.

[R19] Byers-Heinlein K, Fennell CT (2014). Perceptual narrowing in the context of increased variation: Insights from bilingual infants. Developmental Psychobiology.

[R20] Byers-Heinlein K, Jardak A, Fourakis E, Lew-Williams C (2020). Effects of language mixing on bilingual children’s word learning. PsyArXiv.

[R21] Byers-Heinlein K, Lew-Williams C, Fernandez EM, Cairns HS (2018). The handbook of psycholinguistics.

[R22] Carbajal MJ, Peperkamp S (2020). Dual language input and the impact of language separation on early lexical development. Infancy: The Official Journal of the International Society on Infant Studies.

[R23] Casaus GP (2015). The case of bilingual infants.

[R24] Costa A, Sebastián-Gallés N (2014). How does the bilingual experience sculpt the brain?. Nature Reviews Neuroscience.

[R25] De Houwer A, Fletcher P, MacWhinney B (1995). The handbook of child language.

[R26] DeAnda S, Bosch L, Poulin-Dubois D, Zesiger P, Friend M (2016). The language exposure assessment tool: Quantifying language exposure in infants and children. Journal of Speech, Language, and Hearing Research.

[R27] Eimas PD, Siqueland ER, Jusczyk P, Vigorito J (1971). Speech perception in infants. Science.

[R28] ELAN (Version 5.8) [Computer software] (2019). Nijmegen: Max Planck Institute for Psycholinguistics, The Language Archive.

[R29] Estes KG, Evans JL, Alibali MW, Saffran JR (2007). Can infants map meaning to newly segmented words?: Statistical segmentation and word learning. Psychological Science.

[R30] Fennell CT, Waxman SR (2010). What paradox? Referential cues allow for infant use of phonetic detail in word learning. Child Development.

[R31] Ferjan-Ramirez NF, Ramírez RR, Clarke M, Taulu S, Kuhl PK (2017). Speech discrimination in 11-month-old bilingual and monolingual infants: A magnetoencephalography study. Developmental Science.

[R32] Floccia C, Sambrook T, Delle Luche C, Kwok R, Goslin J, White L, Cattani A, Sullivan E, Abbot-Smith K, Krott A, Mills D (2018). Vocabulary of 2-year-olds learning English and an additional language: Norms and effects of linguistic distance. II: Methods. Monographs of the Society for Research in Child Development.

[R33] Flom R (2014). Perceptual narrowing: Retrospect and prospect. Developmental Psychobiology.

[R34] Frank MC, Bergelson E, Bergmann C, Cristia A, Floccia C, Gervain J, Hamlin JK, Hannon EE, Kline M, Levelt C, Lew-Williams C (2017). A collaborative approach to infant research: Promoting reproducibility, best practices, and theory-building. Infancy.

[R35] Friendly RH, Rendall D, Trainor LJ (2014). Learning to differentiate individuals by their voices: Infants’ individuation of native- and foreign-species voices. Developmental Psychobiology.

[R36] Gollan TH, Starr J, Ferreira VS (2015). More than use it or lose it: The number-of-speakers effect on heritage language proficiency. Psychonomic Bulletin & Review.

[R37] Graf Estes K, Bowen S (2013). Learning about sounds contributes to learning about words: Effects of prosody and phonotactics on infant word learning. Journal of Experimental Child Psychology.

[R38] Hoff E (2013). Language development.

[R39] Höhle B, Bijeljac-Babic R, Nazzi T (2020). Variability and stability in early language acquisition: Comparing monolingual and bilingual infants’ speech perception and word recognition. Bilingualism: Language and Cognition.

[R40] Incera S, McLennan C (2018). Bilingualism and age are continuous variables that influence executive function. Aging, Neuropsychology, and Cognition.

[R41] Jasińska KK, Petitto LA (2014). Development of neural systems for reading in the monolingual and bilingual brain: New insights from functional near infrared spectroscopy neuroimaging. Developmental Neuropsychology.

[R42] Kalashnikova M, Pejovic J, Carreiras M (2020). The effects of bilingualism on attentional processes in the first year of life. Developmental Science.

[R43] Kalashnikova M, Singh L, Tsui A, Altuntas E, Burnham D, Cannistraci R, Chin NB, Feng Y, Fernández-Merino L, Götz A, Gustavsson L (2023). The development of tone discrimination in infancy: Evidence from a cross-linguistic, multi-lab report. Developmental Science.

[R44] Kandhadai P, Danielson DK, Werker JF (2014). Culture as a binder for bilingual acquisition. Trends in Neuroscience and Education.

[R45] Kominsky JF (2019). PyHab: Open-source real time infant gaze coding and stimulus presentation software. Infant Behaviour & Development.

[R46] Kovelman I, Salah-Ud-Din M, Berens MS, Petitto L-A (2015). One glove does not fit all” in bilingual reading acquisition: Using the age of first bilingual language exposure to understand optimal contexts for reading success. Cogent Education.

[R47] Kraemer HC, Yesavage JA, Taylor JL, Kupfer D (2000). How can we learn about developmental processes from cross-sectional studies, or can we?. American Journal of Psychiatry.

[R48] Kremin LV, Byers-Heinlein K (2021). Why not both? Rethinking categorical and continuous approaches to bilingualism. International Journal of Bilingualism.

[R49] Kuhl PK, Stevens E, Hayashi A, Deguchi T, Kiritani S, Iverson P (2006). Infants show a facilitation effect for native language phonetic perception between 6 and 12 months. Developmental Science.

[R50] Kuhl PK, Williams KA, Lacerda F, Stevens KN, Lindblom B (1992). Linguistic experience alters phonetic perception in infants by 6 months of age. Science.

[R51] Kuznetsova A, Brockhoff PB, Christensen RHB (2017). lmerTest package: Tests in linear mixed effects models. Journal of Statistical Software.

[R52] Lakens D, Caldwell AR (2021). Simulation-based power-analysis for factorial ANOVA designs. Advances in Methods and Practices in Psychological Science.

[R53] Lakens D, Scheel AM, Isager PM (2018). Equivalence testing for psychological research: A tutorial. Advances in Methods and Practices in Psychological Science.

[R54] Liu L, Kager R (2014). Perception of tones by infants learning a non-tone language. Cognition.

[R55] Liu L, Kager R (2015). Bilingual exposure influences infant VOT perception. Infant Behaviour and Development.

[R56] Liu L, Kager R (2016). Perception of tones by bilingual infants learning non-tone languages. Bilingualism: Language and Cognition.

[R57] Liu L, Kager R (2018). Monolingual and bilingual infants’ ability to use non-native tone for word learning deteriorates by the second year after birth. Frontiers in Psychology.

[R58] Makin T, Xivry JO (2019). Science forum: Ten common statistical mistakes to watch out for when writing or reviewing a manuscript. eLife.

[R59] Maurer D, Werker JF (2013). Perceptual narrowing during infancy: A comparison of language and faces. Developmental Psychobiology.

[R60] Mazuka R, Hasegawa M, Tsuji S (2013). Development of non-native vowel discrimination: Improvement without exposure. Developmental Psychobiology.

[R61] Mercure E, Quiroz I, Goldberg L, Bowden-Howl H, Coulson K, Gliga T, Filippi R, Bright P, Johnson MH, MacSweeney M (2018). Impact of language experience on attention to faces in infancy: Evidence from unimodal and bimodal bilingual infants. Frontiers in Psychology.

[R62] Morgan S, McCloy D (2019). PHOBILE 2.0. Max Plank Institute for the Science of Human History.

[R63] Mousley VL, MacSweeney M, Mercure E (2022). Bilingual toddlers show increased attention capture by static faces compared to monolinguals. Bilingualism: Language and Cognition.

[R64] Narayan CR, Werker JF, Beddor PS (2010). The interaction between acoustic salience and language experience in developmental speech perception: Evidence from nasal place discrimination. Developmental Science.

[R65] Nguyen MVH, Hutchison LA, Norvell G, Mead DL, Winsler A (2023). Degree of bilingualism and executive function in early childhood. Language and Cognition.

[R66] Oakes LM (2010). Using habituation of looking time to assess mental processes in infancy. Journal of Cognition and Development: Official Journal of the Cognitive Development Society.

[R67] Oakes LM, Sperka D, DeBolt MC, Cantrell LM (2019). Habit2: A stand-alone software solution for presenting stimuli and recording infant looking times in order to study infant development. Behaviour Research Methods.

[R68] Orena AJ, Polka L (2019). Monolingual and bilingual infants’ word segmentation abilities in an inter-mixed dual-language task. Infancy.

[R69] Pearson BZ, Fernández SC, Oller DK (1993). Lexical development in bilingual infants and toddlers: Comparison to monolingual norms. Language Learning.

[R70] Pearson BZ, Fernández S, Oller DK (1995). Cross-language synonyms in the lexicons of bilingual infants: One language or two?. Journal of Child Language.

[R71] Peirce JW, Gray JR, Simpson S, MacAskill MR, Höchenberger R, Sogo H, Kastman E, Lindeløv J (2019). PsychoPy2: Experiments in behaviour made easy. Behaviour Research Methods.

[R72] Petitto LA, Berens MS, Kovelman I, Dubins MH, Jasinska K, Shalinsky M (2012). The “Perceptual Wedge Hypothesis” as the basis for bilingual babies’ phonetic processing advantage: New insights from fNIRS brain imaging. Brain and Language.

[R73] Polka L, Masapollo M, Ménard L (2014). Who’s talking now? Infants’ perception of vowels with infant vocal properties. Psychological Science.

[R74] Polka L, Werker JF (1994). Developmental changes in perception of nonnative vowel contrasts. Journal of Experimental Psychology.

[R75] Pons F, Bosch L, Lewkowicz DJ (2015). Bilingualism modulates infants’ selective attention to the mouth of a talking face. Psychological Science.

[R76] Potter CE, Fourakis E, Morin-Lessard E, Byers-Heinlein K, Lew-Williams C (2018). Bilingual toddlers’ comprehension of mixed sentences is asymmetrical across their two languages. Developmental Science.

[R77] Potter CE, Saffran JR (2015). The role of experience in children’s discrimination of unfamiliar languages. Frontiers in Psychology.

[R78] R Core Team (2021). R: A language and environment for statistical computing.

[R79] Ramon-Casas M, Swingley D, Sebastián-Gallés N, Bosch L (2009). Vowel categorization during word recognition in bilingual toddlers. Cognitive Psychology.

[R80] Sebastián-Gallés N, Bosch L (2009). Developmental shift in the discrimination of vowel contrasts in bilingual infants: Is the distributional account all there is to it?. Developmental Science.

[R81] Shook A, Marian V (2012). The bilingual language interaction network for comprehension of speech. Bilingualism: Language and Cognition.

[R82] Singh L, Foong J (2012). Influences of lexical tone and pitch on word recognition in bilingual infants. Cognition.

[R83] Singh L, Loh D, Xiao NG (2017). Bilingual infants demonstrate perceptual flexibility in phoneme discrimination but perceptual constraint in face discrimination. Frontiers in Psychology.

[R84] Singh L, Rajendra SJ, Mazuka R (2022). Diversity and representation in studies of infant perceptual narrowing. Child Development Perspectives.

[R85] Singh T, Tan ARY (2021). Beyond perceptual narrowing: Monolingual and bilingual infants discriminate Hindi contrasts when learning words in the second year of life. Developmental Psychology.

[R86] Streeter LA (1976). Language perception of 2-month-old infants shows effects of both innate mechanisms and experience. Nature.

[R87] Sundara M, Polka L, Molnar M (2008). Development of coronal stop perception: Bilingual infants keep pace with their monolingual peers. Cognition.

[R88] Tomalski P, Malinowska-Korczak A (2020). What do young infants do during eye-tracking experiments? IP-BET - A coding scheme for quantifying spontaneous infant and parent behaviour. Frontiers in Psychology.

[R89] Trehub SE (1976). The discrimination of foreign speech contrasts by infants and adults. Child Development.

[R90] Tsui ASMT, Erickson LC, Mallikarjunn A, Thiessen ED, Fennell CT (2020). Dual language statistical word segmentation in infancy: Simulating a language-mixing bilingual environment. Developmental Science.

[R91] Tyler MD, Best CT, Goldstein LM, Antoniou M (2014). Investigating the role of articulatory organs and perceptual assimilation in infants’ discrimination of native and non-native fricative place contrasts. Developmental Psychobiology.

[R92] Werker JF (2012). Perceptual foundations of bilingual acquisition in infancy. Annals of the New York Academy of Sciences.

[R93] Werker JF (2018). Perceptual beginnings to language acquisition. Applied Psycholinguistics.

[R94] Werker JF, Byers-Heinlein K, Fennell CT (2009). Bilingual beginnings to learning words. Philosophical Transactions of the Royal Society B: Biological Sciences.

[R95] Werker JF, Byers-Heinlein K (2008). Bilingualism in infancy: First steps in perception and comprehension. Trends in Cognitive Sciences.

[R96] Werker JF, Curtin S (2005). PRIMIR: A developmental framework of infant speech processing. Language Learning and Development: The Official Journal of the Society for Language Development.

[R97] Werker JF, Gilbert JH, Humphrey K, Tees RC (1981). Developmental aspects of cross-language speech perception. Child Development.

[R98] Werker JF, Tees RC (1984). Cross-language speech perception: Evidence for perceptual reorganization during the first year of life. Infant Behaviour & Development.

[R99] Werker JF, Yeung HH, Yoshida KA (2012). How do infants become experts at native-speech perception?. Current Directions in Psychological Science.

[R100] Zadina JN (2015). The emerging role of educational neuroscience in education reform. Psicología Educativa.

